# Comment on “Optic Nerve Sheath Diameter Ultrasound Evaluation in Intensive Care Unit: Possible Role and Clinical Aspects in Neurological Critical Patients' Daily Monitoring”

**DOI:** 10.1155/2018/6154357

**Published:** 2018-09-16

**Authors:** Maddalena De Bernardo, Nicola Rosa

**Affiliations:** Department of Medicine Surgery and Dentistry “Scuola Medica Salernitana”, University of Salerno, Italy

We read with great interest the article by Toscano et al. [1] concerning the possible role and clinical aspects of ultrasound evaluation in intensive care unit.

We would like to congratulate the authors for their interesting paper, but we would like to make some comments because we are a little bit concerned with the way they establish the presence of increase in intracranial pressure (ICP).

We strongly disagree with the authors when they concluded that optic nerve sheath diameter (ONSD) evaluation with ultrasound can be performed with short training of almost every medical and paramedical personnel.

When we deal with ocular structure, to get precise measurements is mandatory, because few microns can make a difference [2].

To make a precise measurement of the optic nerve diameter is not a simple task. First of all, when the ONSD is measured with B scan, several mistakes can be made, as we clarified in several reports [3].

Moreover a precise knowledge of the anatomy and principles of echography have to be known; otherwise mistakes can be present.

For example, if we consider Figures 1(b) and 1(c) that should show the best example of the measurements taken, there are several flaws. It clearly appears that the magnification of the pictures is different, being higher in the picture on the right, giving the impression that the ON is even wider. This is proven by the length of the yellow line that is clearly longer compared to the first.

The two markers of the yellow lines, which should prove that the measurements are taken at 3 mm from the insertion, are positioned differently: in Figure 1(c) one starts at the optic disc level, but in Figure 1(b) the same marker starts far inside the vitreous body, proving that the measurements are not taken at the same level.

Furthermore, the position of the markers is not at the level of the interface between fluid and arachnoid.

These pictures clearly prove that when measuring the ONSD a bias can be present, which could significantly influence the results.

It is true that ultrasound can be used to detect in real time changes in the intracranial pressure; however, to get reliable measurement, standardized A scan, which is less influenced by personal errors but needs a skill, should be utilized [4–6].

With this technique, utilizing the so called 30° test, that proves the presence of subarachnoidal fluid in case of OSND decrease greater than 5% during maximal abduction of the eye, it is also possible to differentiate an increased ICP from solid thickening or swelling of the pial and arachnoidal sheaths due to optic neuritis, ON glioma, meningioma, or leukemic infiltration [6].

## References

[1] Toscano M., Spadetta G., Pulitano P. (2017). Optic Nerve Sheath Diameter Ultrasound Evaluation in Intensive Care Unit: Possible Role and Clinical Aspects in Neurological Critical Patients’ Daily Monitoring. *BioMed Research International*.

[2] Rosa N., De Bernardo M., Lanza M. (2013). Axial eye length evaluation before and after hyperopic photorefractive keratectomy. *Journal of Refractive Surgery*.

[3] De Bernardo M., Rosa N. (2017). Clarification on using ultrasonography to detect intracranial pressure. *JAMA Ophthalmology*.

[4] Ossoinig K. C., Cennamo G., Frazier-Byrne S. (1981). Echographic Differential Diagnosis of Optic-Nerve Lesions. *Ultrasonography in Ophthalmology*.

[5] Rosa N., De Bernardo M. (2017). Measurement of the optic nerve in a resource-limited setting. *Journal of Neurosciences in Rural Practice*.

[6] Ossoinig K. C. (1990). Standardized echography of the optic nerve. *Ophthalmic Echography 13*.

